# Incidence and prevalence of mucous membrane pemphigoid with ocular involvement: a retrospective analysis using the TriNetX database

**DOI:** 10.1038/s41433-025-04040-5

**Published:** 2025-10-08

**Authors:** Camellia Edalat, Matthew Spangler, Jennifer Thorne, Paulina Liberman, Meghan Berkenstock

**Affiliations:** 1 The Drexel University College of Medicine, Philadelphia, PA USA; 2 Division of Ocular Immunology, The Wilmer Eye Institute, the Johns Hopkins University School of Medicine, Baltimore, MD USA; 3 Center for Clinical Trials, Bloomberg School of Public Health, Baltimore, MD USA

**Keywords:** Epidemiology, Autoimmune diseases

## Abstract

**Background:**

Mucous membrane pemphigoid with ocular involvement (oMMP) is an autoimmune disease that results in chronic conjunctivitis, conjunctival scarring, and if left untreated, permanent vision loss. oMMP is quite rare with incidence rates between one in 12,000 to one in 60,000, but there is a lack of large population-based studies that focus solely on oMMP. Thus, we sought to examine the cumulative and annual incidence and prevalence of oMMP in the TriNetX database and compared these findings to the US population for greater generalizability.

**Methods:**

This was a retrospective study utilised International Classification of Disease, 10th edition (ICD-10) codes to determine the yearly and cumulative incidence and prevalence, demographics, ocular complications, and immunosuppressant treatments prescribed for oMMP from 2013 to 2023. TriNetX software was used to analyze the data.

**Results:**

A total of 4052 patients were diagnosed with oMMP with a mean age of 73 years (SD = 14; range 18–90). The majority of patients were female (n = 2604 64.26%) and non-Hispanic, white (n = 3098, 76.46%). Prednisone was the most common systemic medication prescribed to 40% of patients. The most used immunosuppressive drug was mycophenolate mofetil (21%). Glaucoma was the most common ocular complication (n = 375, 9.25%). Between 2013 and 2023, there was a continuous rise in the cumulative incidence and prevalence rates. The 11-year cumulative incidence and prevalence were 1.41per 100,000 persons and 6.72 per 100,000 persons, respectively.

**Conclusions:**

Compared to current literature, the TriNetX population with oMMP had similar demographics and immunosuppressant use, but showed a higher incidence and an overall increasing prevalence.

## Introduction

Mucous membrane pemphigoid with ocular involvement (oMMP) is an autoimmune disease that results in chronic conjunctival inflammation and scarring [1]. Without treatment, it can lead to permanent vision loss [1]. The pathogenesis of oMMP is characterised by an autoimmune response resulting in the deposition of immunoglobulins and complement components at the basement membrane of the conjunctiva, triggering an inflammatory cascade [2]. Autoantibodies target conjunctival basement membrane antigens such as laminin 332, collagen VII, BP180, and α6β4 integrin [3]. Because of the risk of blindness and permanent damage to the cornea, oMMP is considered to be higher risk disease with more aggressive therapy indicated compared to patients with mucous membrane pemphigoid (MMP) and only oral, nasal, or skin involvement [4, 5].

Definitive diagnosis of oMMP requires demonstrating linear deposits of immunoglobulin or complement at the conjunctival basement membrane zone [6]. However, it should be noted that if clinical findings support an isolated oMMP, the diagnosis can be made provided that other differential diagnoses were ruled out based on clinical findings and histology. Systemic immunosuppressive therapy is the cornerstone of management due to the sight-threatening nature of the disease [7]. Cyclophosphamide, combined with prednisone, has been utilised for severe or rapidly progressing oMMP [3, 8]. This combination has been shown to achieve high rates of disease control and remission, but it must be used with caution due to adverse effects [9–11]. Rituximab, a monoclonal antibody targeting CD20 on B cells, has emerged as an effective treatment for oMMP. Studies have shown that rituximab can achieve high rates of complete response and sustained remission [12, 13] Early aggressive treatment with rituximab has demonstrated corticosteroid-sparing success in up to 81.2% of patients, with no significant progression of the cicatrising disease or worsening of visual acuity [11, 14–16]. A more conservative treatment strategy suggests using a stepladder approach, starting with antimetabolites (typically mycophenolate mofetil or methotrexate) with escalation to biologics and cyclophosphamide in severe cases or those recalcitrant to treatment [11].

Given the rarity of the disease, incidence rates of oMMP vary between one in 12,000 to one in 60,000 persons depending on the population studied [13]. However, disease findings may be subtle and overlooked at earlier stages leading to an underestimation of the incidence. Furthermore, there is a lack of large population-based studies that focus solely on oMMP [17, 18]. The TriNetX Collaborative network is a large database of de-identified data from 65 US health care networks with a population of over 109 million patients. We sought to examine the cumulative and annual incidence and prevalence of oMMP in the TriNetX database along with the demographics and immunosuppressive drug use among these patients.

## Methods

### Patient Cohorts

TriNetX utilises the International Classification for Diseases, 10^th^ Edition (ICD-10) codes to identify and analyse patient cohorts. The mucous membrane pemphigoid with ocular involvement cohort was identified using the ICD-10 code L12.1. When ICD9 codes were in use, oMMP diagnosis required coding for chronic conjunctivitis and mucus membrane pemphigoid. However, ICD9 codes were converted to the corresponding ICD10 codes in the TriNetX database utilising General Equivalence Mappings created by the Centers for Medicare & Medicaid Services and the Centers for Disease Control and Prevention. Data were collected in March 2024 and included sex, age (greater than or equal to 18 years old), race, associated immunosuppressive agents, ocular complications, and the yearly incidence and prevalence.

### Immunosuppressive medication trends

Immunosuppressive medications examined for the management of oMMP were collected using RxNorm numbers. The queries were collected using a time constraint of medication being prescribed at or after the diagnosis of oMMP. The medications examined were: prednisone (8640), dapsone (3108), methylprednisolone (6902), mycophenolate mofetil (265323), prednisolone (8638), cyclosporine (3008), rituximab (121191), tacrolimus (42316), methotrexate (6851), azathioprine (1256), mycophenolic acid (7145), cyclophosphamide (3002), and infliximab (191831).

### Ocular complications

The oMMP cohort was further cross-queried with macular oedema (H35.35x, H35.81x), glaucoma (H40.1x, H40.2x, H40.4x, H40.5x, H40.6x, H40.8x, H40.9x), blindness and low vision (H54x), corneal scarring (H17.9), cicatricial entropion (H02.01), trichiasis (H02.05), cicatricial ectropion (H02.11), and cicatricial lagophthalmos (H02.21). A time constraint was included ensuring that the ocular event occurred at the same time or after the diagnosis of oMMP.

### Incidence and prevalence

The incidence and prevalence of oMMP were calculated and analysed using the TriNetX analytics model from January 1, 2013, through December 31, 2023. The data were collected as yearly values in cases per 100,000 persons to examine the trends over the 11-year time period and determine the cumulative incidence and prevalence.

### About the TriNetX database

This study utilised the US Collaborative Network with natural language processor (NLP). NLP is a software employed by TriNetX to encode written patient information from physician notes and clinical reports into ICD-10 coded inputs. With greater advancements in NLPs, they are increasing in their accuracy and validity when it comes to identifying rare disease [19]. De-identification of the patient data follows Section >>164.514(b)(C) of the Health Insurance Portability and Accountability Act Privacy Rule and has been attested by qualified experts. Due to TriNetX databases lack of patient-identifiable information, this paper was deemed exempt by the Drexel University College of Medicine Institutional Review Board (IRB) and was compliant with the Declaration of Helsinki.

## Results

### Demographics

A total of 4052 patients with oMMP were identified with an average age of 73 years (SD = 14; range 18–90) and who were 64.26% female (n = 2604), 78.72% white (n = 3190) and 76.46% non-Hispanic or Latino (n = 3098). Demographics are found in Table 1.

**Table 1:** Demographics of subjects with mucous membrane pemphigoid with ocular involvement in the TriNetX database.

Total patients	4052
Age	
Min	18
Max	90
Mean	73
Standard Deviation	14
Gender (%)	
Female	2604 (64.26)
Male	1447 (35.71)
Unknown	1 (0.03)
Ethnicity (%)	
Not Hispanic or Latino	3098 (76.46)
Unknown Ethnicity	845 (20.85)
Hispanic or Latino	109 (2.69)
Race (%)	
White	3190 (78.72)
Unknown Race	429 (10.58)
Black	190 (4.68)
Other Race	132 (3.25)
Asian	91 (2.24)
Native Hawaiian	13 (0.32)
American Indian	8 (0.21)

Demographics of patients with mucous membrane pemphigoid with ocular involvement.

### Immunosuppressants and corticosteroids

The most commonly used immunosuppressive drugs (Table 2) were prednisone (40%), methylprednisolone (28%), and mycophenolate mofetil (21%). Cyclophosphamide and infliximab were the least commonly used immunosuppressants (2% and 1%, respectively).

**Table 2:** Immunosuppressants used to treat subjects with mucous membrane pemphigoid with ocular involvement in the TriNetX database.

	N	%
Total patients	4052	100%
Prednisone	1633	40%
Methylprednisolone	1150	28%
Mycophenolate mofetil	870	21%
Prednisolone	743	18%
Dapsone	711	18%
Cyclosporine	495	12%
Rituximab	452	11%
Tacrolimus	367	9%
Methotrexate	357	9%
Azathioprine	291	7%
Mycophenolic acid	189	5%
Cyclophosphamide	83	2%
Infliximab	24	1%

Usage of immunosuppressants for treating patients with oMMP.

### Ocular complications

Glaucoma was the most common ocular complication among oMMP patients (n = 375, 9.25%), followed by low vision and blindness (n = 295, 7.28%), cicatricial entropion (n = 286, 7.06%), and corneal scarring and trichiasis (n = 223, 5.5%). Cicatricial ectropion (n = 67, 1.65%) and cicatricial lagophthalmos (n = 14, 0.34%) were the lowest reported complications.

### Incidence and prevalence

**Fig. 1:**
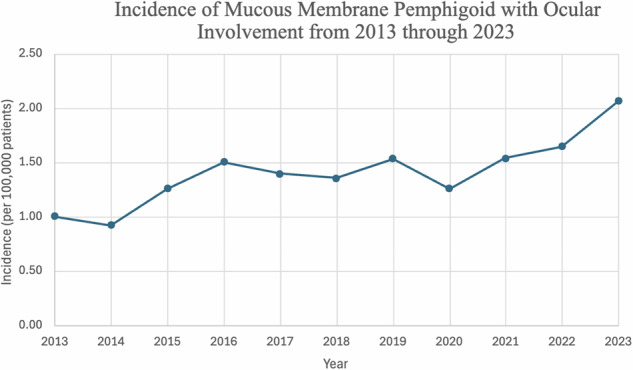
Incidence of patients with oMMP from 2013 to 2023.

**Fig. 2:**
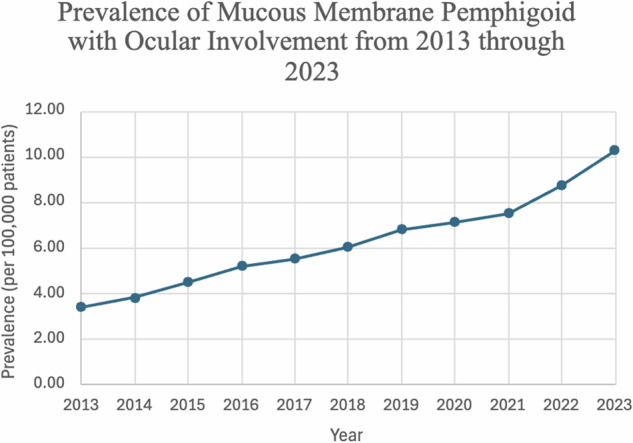
Prevalence of patients with oMMP from 2013 to 2023.

The cumulative prevalence was 6.72 per 100,000 persons, and the cumulative incidence rate was 1.41 per 100,000 persons over the 11-year period. Incidence increased over time from 1.00/100,000 in 2013 to 2.06/100,000 in 2023 (Fig. 1) with a drop in 2020. With the exception of three slight decreases in the yearly incidence, the first in 2014 (1.00/100,000 in 2013 to .92/100,000 in 2014), the second in 2016 (1.50/100,000 in 2016 to 1.36/100,000 in 2018), and the third from 2019 into 2020 (1.53/100,000 in 2019 to 1.26/100,000), there was continuously increasing incidence and prevalence throughout the 11-year period to 10.3/100,000 in 2023 (Fig. 2). The cumulative incidence and prevalence for females was 1.58/100,000 and 7.83/100,000, respectfully, which was higher than that of males (1.18/100,000; 5.39/100,000). Individuals who were not of Hispanic or Latino origin had a cumulative incidence of 1.57/100,000 compared to 0.49/100,000 in those who were of Hispanic or Latino origin. A similar trend was seen among their cumulative prevalence as well, which was 8.81/100,000 versus 2.31/100,000. White patients had the highest incidence and prevalence throughout the 11-year period (1.64/100,000, 8.55/100,000). Native Hawaii and other Pacific Islanders had the lowest cumulative incidence at 0.42/100,000, while Black or African American patients had the lowest cumulative prevalence at 2.30/100,000 (Table 3). The overall incidence of patients under the age of 70 was 1.47/100,000 compared to 1.54/100,000 in patients aged 70 and older. Prevalence followed the same pattern with 7.46/100,00 in the under 70 cohort and 8.05/100,000 in the 70 s and over.

**Table 3:** Incidence and prevalence of mucous membrane pemphigoid with ocular involvement stratified by race.

Race	Incidence (per 100,000)
American Indian or Alaska Native	1.22
Asian	0.68
Black or African American	0.44
Native Hawaiian or Other Pacific Islander	0.42
White	1.64
Other Race	1.09
Unknown Race	2.07

Race	Prevalence (per 100,000)
American Indian or Alaska Native	4.85
Asian	3.26
Black or African American	2.30
Native Hawaiian or Other Pacific Islander	2.56
White	8.55
Other Race	3.77
Unknown Race	5.75

Incidence and prevalence of oMMP stratified by race.

## Discussion

The current literature is extremely limited in its epidemiologic analysis of oMMP, with case studies comprising the bulk of the publications due to the rarity of the condition. Larger studies, mainly based out of Europe, have indicated varying incidence rates of 1 in 12,000 to 1 in 60,000 with no reports on prevalence [20, 21]. One study focused on the incidence and prevalence of oMMP in Colombia utilising the national health registry, and reported an average incidence of 0.24 per 1,000,000 individuals and an average prevalence of 0.22 per 1,000,000 [22]. The use of TriNetX limited to the population in the US allows for a culturally diverse group for analysis, despite the rarity of the condition, and a larger sample size for a more robust assessment of the incidence and prevalence results. Our study found an increasing incidence and prevalence across the 11-year period, which could not only be attributed to rising cases of the disease but also increased awareness of its presentation, stronger data collection, and overall better diagnosis.

The treatment of oMMP is focused on halting the progression of fibrosis and controlling inflammation [3]. Systemic immunosuppressive drug therapy is the gold standard as topical treatment alone is insufficient and ineffective [23]. The choice of therapy often depends on the disease stage and severity. Most patients present with moderate to advanced disease, requiring aggressive treatment with biologics (such as rituximab) or cyclophosphamide [11, 24]. Our study found that corticosteroids (prednisone and methylprednisolone) and mycophenolate mofetil were the most commonly used agents prescribed for oMMP management in the TriNetX database. This finding could indicate that the majority of patients within the cohort had milder disease, were being trialled on antimetabolites prior to escalation of therapy, or that ophthalmologists felt more comfortable prescribing mycophenolate comparted to other immunosuppressive agents [25]. Although these results align with the more conservative literature supporting antimetabolite therapy as a first-line approach, we favour early introduction of more aggressive therapy, particularly biologics such as rituximab [10, 14].

The most common ocular complication in our study was glaucoma. This differed from a systematic review and meta-analysis by Bocanegra-Oyola et al., which found that entropion and trichiasis were the most frequent complications reported [26]. In our dataset, entropion and trichiasis were the fourth most common complications behind low vision and blindness and corneal scarring. This discrepancy, again, can correlate with a population with milder disease. Previous research shows that severe oMMP often leads to corneal scarring, vision loss, persistent epithelial defects, and limbal inflammation, which in turn causes keratopathy and visual impairment. This suggests that variations in ocular complications across studies are directly related to disease severity and stage at the time of evaluation [4, 7, 24].

There was a notable decrease in the incidence in 2020, likely related to the SARS-CoV-2 pandemic with less presentation of patients for diagnosis due to clinic closures and reduced provider schedules [27, 28]. Beyond this, the fluidity of the TriNetX database could lead to small deviations in the prevalence, as noted in 2014, 2017, and 2018. This also highlights several limitations to our study. First, the retrospective cohort design is entirely reliant on accurate coding and consolidation of data from multiple electronic medical records (EMR) into the TriNetX database. TriNetX provides a comprehensive dataset; however, it is dynamic due to the constant addition of new healthcare organisations. The integration of data from these new entities may influence both prevalence and incidence rates over time, as the expanding dataset captures a broader and more diverse patient population. This dynamic nature can introduce variability in the observed trends, making it challenging to attribute changes solely to temporal factors rather than data source expansion. Secondly, due to the anonymised and aggregated nature of the data, we cannot manually review charts to assess for accurate coding. Beyond this, the presence of a singular ICD-10 code for oMMP limited our ability to further analyse the patterns of the disease based on its varying degrees of severity, determine whether patients had a positive histopathology result, and evaluate for more intricacies within complication groups. Furthermore, we were unable to assess whether the diagnosis was coded by an ophthalmologist versus a dermatologist in the greater context of systemic oMMP due to the nature of the database. Additionally, due to the use of ICD-10 codes, we were unable to further specify their definitions, such as ‘low vision and blindness’. From here, we must also discuss the potential of false positives and false negatives when it comes to disease diagnosis. OMMP is uncommon and under-coded, which could contribute to disparities in true diagnosis. Additionally, with the use of the database with an NLP, symptoms could be missed when aggregating information, or a diagnosis could be counted as positive in the context of duplicate records. Finally, with the nature of this study, there are inherent biases that may be present. Health record data may not accurately capture the patient’s true health status, and any Artificial Intelligence algorithms configured on this data can then amplify these existing biases.

## Conclusion

Our results suggest a higher incidence of oMMP than previously reported. Data from TriNetX also show that corticosteroids and mycophenolate mofetil were the most commonly prescribed treatments for oMMP. This treatment pattern could point to a predominance of milder disease within the cohort, earlier intervention in the disease process, or the utilisation of a stepwise approach in managing the condition.

## Summary

### What was known before

- Diagnosis and management of mucous membrane pemphigoid with ocular involvement has been heavily discussed in current literature, however information on its true incidence and prevalence are lacking due to minimal sample sizes available in studies.

### What this study adds

- Our study found a greater incidence and prevalence than previously reported, as well as demographic, immunosuppressant, and ocular complication trends, based off a much larger sample size.
- These findings provide a more generalisable number to the disease thus indicating a need for earlier screening and management to avoid vision threatening complications.

## Supplementary information

Eye Reporting Checklist

## Data Availability

The data that support the findings of this study are available from the TriNetX platform but restrictions apply to the availability of these data, which were used under license for the current study, and so are not public available. Data are however available from the authors upon reasonable request and with permission of TriNetX.
